# Evaluation of antiviral activity of *Bacillus licheniformis*-fermented products against porcine epidemic diarrhea virus

**DOI:** 10.1186/s13568-019-0916-0

**Published:** 2019-12-03

**Authors:** Ju-Yi Peng, Yi-Bing Horng, Ching-Ho Wu, Chia-Yu Chang, Yen-Chen Chang, Pei-Shiue Tsai, Chian-Ren Jeng, Yeong-Hsiang Cheng, Hui-Wen Chang

**Affiliations:** 1 Graduate Institute of Molecular and Comparative Pathobiology, School of Veterinary Medicine, National Taiwan University, No. 1, Section 4, Roosevelt Rd., Taipei, 10617 Taiwan; 2 School of Veterinary Medicine, National Taiwan University, No. 1, Section 4, Roosevelt Rd., Taipei, 10617 Taiwan; 3 Department of Biotechnology and Animal Science, National Ilan University, No. 1, Sec. 1, Shennong Rd., Yilan County, Yilan City, 260 Taiwan; 4 Institute of Veterinary Clinical Science, School of Veterinary Medicine, National Taiwan University, Taipei, 10617 Taiwan

**Keywords:** *B. licheniformis*-fermented products, PEDV, Antiviral

## Abstract

*Bacillus licheniformis* (*B. licheniformis*) is commonly used as probiotic and its secondary metabolites are attractive anti-microbial candidate. In the present study, we aimed to evaluate the antiviral activity of crude extracts from *B. licheniformis* against porcine epidemic diarrhea virus (PEDV), a highly contagious enveloped porcine virus that has caused great economic loss in pigs. In vivo, PEDV-infected piglets supplemented with air-dried solid state fermentative cultivate containing *B. licheniformis*-fermented products (BLFP) showed milder clinical symptoms and decreased viral shedding. Importantly, no significant systemic pathological lesions and no reduction in average daily gain were noted in pigs supplemented with the BLFP, which suggests that it is safe for use in pigs. In vitro experiments revealed that while *B. licheniformis* crude extracts exhibited no toxicity in Vero cells, co-cultivation of *B. licheniformis* crude extracts with PEDV significantly reduced viral infection and replication. Summarized current results suggest that the *B. licheniformis*-fermented products could be a novel candidate food additive for reducing the impact of PED on the swine industry.

## Introduction

Beginning in 2013, outbreaks of new variants of porcine epidemic diarrhea virus (PEDV) have caused high mortality and morbidity in piglets, leading to great economic losses (Lee 2015). The virus causes porcine epidemic diarrhea (PED), which is characterized by watery diarrhea, vomiting, and severe dehydration in pigs of all ages, especially suckling piglets (Lee 2015; Song and Park 2012). This highly contagious disease has spread quickly in porcine industries in several countries (Jung and Saif 2015; Lee 2015). Furthermore, PEDV infection causes severe perturbations of gut microbiota, reducing probiotic bacterial abundance, enriching pathogenic bacteria, and even impairing the growth performance of PEDV-surviving pigs (Alvarez et al. 2015; Deping Song et al. 2017). The development of effective protective agents against PEDV infection is urgently needed.

Probiotics is one of the choices to be an alternative to antibiotic growth promoter, AGP (Abudabos et al. 2017). Our recent studies reported that *Lactobacillus* species and *Clostridium butyricum*-fermented probiotics product alleviate diarrhea incidence and reduce the gut pathogens in weaning piglets (Cheng et al. 2019). On the other hand, *Bacillus subtilis* (*B. subtilis*) and *B. licheniformis*–fermented products could increase growth performance and mitigate *Clostridium perfringens*–induced necrotic enteritis in broilers (Cheng et al. 2018; Lin et al. 2019). Enhancement of nutrient digestibility and *Lactobacillus* counts of feces by feeding *B.* subtilis and *B. licheniformis* has also been reported in pigs (Lan and Kim 2019). It has been demonstrated that *B. licheniformis* exhibiting antimicrobial activity against pathogens might be due to the production of antibacterial biosurfactants (Lin et al. 2019). Furthermore, our experiment results also demonstrated that *B. licheniformis*–fermented products exhibit antibacterial activities against *Clostridium perfringens* and *Staphylococcus aureus* in vitro (Lin et al. 2019).

In the present study, the antiviral effect of the BLFP crude extract against PEDV were evaluated in pigs. The surfactin-like peptide in the BLFP crude extract was identified from the secondary metabolite of *B. licheniformis* fermentative cultivates. The in vitro toxicity and antiviral ability of the surfactin-like peptide in the BLFP crude extract against PEDV were evaluated using the Vero cells.

## Materials and methods

### Cells and viruses

Vero C1008 cells (American Type Culture Collection (ATCC) No. CRL-1586) were maintained in DMEM (Gibco, NY, USA) supplemented with 10% fetal bovine serum (Hyclone, Utah, USA) and 1% penicillin–streptomycin-amphotericin B (Gibco, NY, USA). The passage 6 PEDV-Pintung 52 strain (PEDVPT-P6) viral stock was used at a titer of 1 × 10^5^ TCID_50_/mL.

### Extraction and quantification of biosurfactants from BLFP crude extract

Air-dried solid-state fermentative cultivates of *B. licheniformis* (Weigmann) Chester (ATCC^®^27811™) were suspended in distilled water and heated at 30 °C for 30 min. Supernatants were harvested after centrifugation at 13,000 rpm for 30 min. After adjusting the pH value to 2.0 with 6 N HCl for protein precipitation, the precipitates were dried in a freeze dryer after washing twice with distilled water. This *B. licheniformis*-*fermented products* (BLFP) was used in animal study. To characterized the *BLFP*, the surfactin derived from *Bacillus subtilis* (Sigma-Aldrich, St Louis, USA) was used as standard substance. The content of BLFP in the fermentative product was determined by high-performance liquid chromatography (HPLC) as previously described (Schneider and Marahiel 1998). Briefly, after filtration, samples were subjected to analysis via SPD-10A HPLC (Shimadzu, Japan) with a pre-packed LiChrospher 100 RP-18 column (Merck, Darmstadt, Germany). The mobile phase was a mixture of 3.8 mM trifluoroacetic acid and 200 ml DDW with 800 ml 100% methanol. Elution was performed at a flow rate of 1 ml/min and determined with a UV detector (10A VP, Shimadzu, Tokyo, Japan) at 210 nm. The gradient strategy was as follows: 0–3.5 min, 60% A to 93% A; 3.5–20 min, keeping 93% A and 7% B (A, acetonitrile; B, ultrapure water); min pressure 0 bar, max pressure 400 bar, pressure stability 10 bar; injection volume 10 μl; syringe speed 8 μl/s; flush volume 800 μl. Liquid chromatography–mass spectrometry (LC–MS) full scan positive mode was performed with *m/z* ranging from 200 to 2000.

### Animals and study design

Fifteen 4-week-old, PEDV-fecal RNA and PEDV seronegative, Large White × Duroc crossbred pigs were acquired from a conventional pig farm with no known history of PED. Treatments were: (1) Control (*n *= 5); (2) PEDV (*n *= 5); and (3) PEDV + BLFP (n = 5). These pigs were fed a commercial diet mixed with or without 5 kg/L BLFP as feed additives for 7 days prior to the viral challenge, and were challenged with or without 5 × 10^5^ TCID_50_ of the virulent PEDVPT-P6 (P6) at 5 weeks of age (Table 1). Each group was housed in a separate fenced area. Clinical symptoms, fecal consistency scoring, and fecal viral shedding were recorded and tested daily. The clinical scores were recorded using a 4-scale: 0, normal feces; 1, pasty; 2, semi-fluid; 3, fluid. All piglets were weighed weekly and sacrificed 19 days post-infection (DPI) for safety assessment by pathological examination. The animal experiment was approved by the Institutional Animal Care and Use Committee of National Taiwan University (Taipei, Taiwan, NTU105-EL-00087).

**Table 1:** Animal experimental design

Group	Number	BLFP	Virus challenge
Control	5	0	0
PEDV + BLFP	5	5 kg/ton feed	PEDVPT-P6 (oral 5 × 10^5^ TCID_50_)
PEDV	5	0	PEDVPT-P6 (oral 5 × 10^5^ TCID_50_)

BLFP, *Bacillus licheniformis*-fermented products

### Real-time quantitative RT-PCR

Real-time quantitative RT-PCR (qPCR) was performed based on a previously described method with modification (Chang et al. 2017). The viral RNA was extracted from 200 μl of culture supernatants using Cador^®^ Pathogen 96 QIAcube^®^ (Qiagen, Chatsworth, CA, USA) according to the manufacturer’s instructions. Reverse transcription was performed using the QuantiNova Reverse Transcription Kit (Qiagen) according to the manufacturer’s protocol. For qPCR assay, the QuantiNova Probe Master Mix (Qiagen, Chatsworth, CA, USA) was used with the following primers and probe for PEDV detection: PEDV FN: 5′-CGCAAAGACTGAACCCACTAA C-3′; PEDV RN: 5′-TTGCCTCTGTTGTTACTTGGAGAT-3′; and probe: 5′-FAM-TGYYACCAYYACCACGACTCCTGC-BHQ1. qPCR was performed under following conditions: 95 °C for 2 min, then 45 cycles of 95 °C for 15 s and 55 °C for 15 s. The averaged cycle threshold (Ct) values of three replicates of extracellular supernatants and five replicates of fecal swabs were used to determine the genomic equivalents (GE). The detection limit of the real-time RT-PCR was 60 GE of DNA using the plasmid encoding the PEDV N gene as a standard (data not shown).

### Gross and histopathological examination

All pigs were euthanized and necropsy was performed at 19 DPI to evaluate the safety of BLFP in pigs. Representative tissue samples were collected, fixed in 10% neutral-buffered formalin, processed routinely, sliced into 5-μm-thick sections, and stained with hematoxylin and eosin. The histopathological observations were recorded and assessed blindly by one veterinary pathologist.

### In vitro cytotoxicity assay of BLFP crude extract

To evaluate the cytotoxicity of BLFP crude extract in vitro, Vero cells were first grown in a 96-well microplate (Corning Life Sciences, Corning, NY, USA) at a density of 20,000 cells per well 1 day prior to the experiment. After removing the culture supernatant, ten-fold serially diluted BLFP crude extract in PI medium (DMEM supplemented with 0.3% tryptose phosphate broth (Sigma-Aldrich, Mo, USA), 0.02% yeast extract (Acumedia, Michigan, USA), and 10 μg/ml of trypsin (Sigma-Aldrich, Mo, USA) was added to the cell monolayer. After 48 h, the culture supernatant was removed and 100 μl AlamarBlue™ was added (G-Biosciences, St. Louis, USA; 10% in PBS). After 2 h of incubation at 37 °C, the plate was read with the excitation wavelength at 575 nm and with the emission wavelength at 590 nm. The OD value of each well was calculated to obtain the percentage reduction of alamarBlue according to the manufacturer’s instructions. Additionally, an untreated group G1, which consisted of Vero cells without any treatment, was included to represent 100% reduction of alamarBlue. All data were further calculated as a percentage of the untreated group G1. Normalized data were plotted against concentrations of BLFP crude extract and fitted to a non-linear regression curve using GraphPad Prism (GraphPad Software, San Diego, CA). The 50% cytotoxicity concentration (CC_50_, the concentration of BLFPat which cellular viability was reduced to 50%) was calculated accordingly.

### Time-of-addition assay of BLFP crude extract

To study the antiviral activity of BLFP crude extract against PEDV, the biosurfactants were added at different time points during the viral infection. Vero cells were seeded in a 96-well microplate (20,000 cells/well) 1 day before the experiment. BLFP crude extract at 150 ppmin PI medium were added to each well in different orders to treat 200 TCID_50_/ml PEDV infections in cells. The experiment included five groups (as illustrated in Fig. 4a): the virus treated group (G2) contained Vero cells treated with the solvent of BLFP crude extract; the pre-treatment group (G3) contained Vero cells treated with the BLFP crude extract 1 h prior to PEDV infection; the co-treatment group (G4) contained Vero cells treated with BLFP crude extract at the same time as PEDV infection; the post-treatment group (G5) contained Vero cells treated with BLFP crude extract 1 h post PEDV infection. In G2-G5, culture supernatants were removed and replaced with fresh PI medium after 1 h of viral infection or BLFP crude extract treatment. In addition, the post-drug group (G6) contained Vero cells treated with BLFP crude extract 1 h post PEDV infection and kept in the culture medium for the entire 48 h of the study. After 48 h, the supernatants of all groups were harvested to determine the virus titer and viral load, and cellular viability was examined using the AlamarBlue™ assay as descried above.

### Replication kinetics assay of PEDV in cells with or without BLFP crude extract

To further investigate the antiviral mechanism in the post-drug group (G6), the replication kinetics of PEDV in Vero cells with or without crude extract of BLFP were compared. Vero cells were seeded in a 96-well microplate (20,000 cells/well) 1 day before the experiment. The crude extract of BLFP at 150 ppm in PI medium was added to 200 TCID_50_/ml PEDV-infected cells. Culture supernatants and cells were collected separately at the 2, 8, 12, 24, and 48 h post-infection. Viral titers in culture supernatants were determined using the Reed-Müench method (Reed 1938) and are expressed as the 50% TCID_50_/ml. Virus-specific RNA in culture supernatants and in cells was quantified by real-time reverse transcription-PCR.

### Determination of PEDV TCID_50_

Virus titration was performed in the culture supernatants. Briefly, the first 10-fold dilution and the subsequent 10-fold serial dilution of the supernatants were added to 20,000 cells/well Vero cells on 96-well microplates. After 1 h of incubation, the inoculants were aspirated, and the cells were washed with PI medium two times and then cultured in PI medium. After 72 h, the plate was subjected to cytopathic effect (CPE) observation and the titer of PEDV was determined using the Reed-Müench method (Reed 1938) and is expressed as the 50% TCID_50_/ml.

### Determination of IC_50_ of the BLFP crude extract

To determine the IC_50_, Vero cells were seeded in 96-well microplates (20,000 cells/well) one day before the experiment. Ten-fold serially diluted crude extract of BLFP in PI medium was added to Vero cells 1 h after 200 50% tissue culture infectious dose (TCID_50_) PEDV infection. After 48 h, the percent reduction of alamarBluestaining was examined by the AlamarBlue™ assay as described above. All normalized data were plotted and fitted to a nonlinear regression curve in GraphPad Prism (GraphPad Software) to generate the IC_50_.

### Direct virucidal activity of BLFP crude extract

To elucidate the direct virucidal activity of crude extract of BLFP against PEDV, the PEDVPT-P6 viral stock at a titer of 1 × 10^5^ TCID_50_/ml was mixed with or without 150 ppm BLFP for 1 h at 4 °C. The mixtures were serially diluted 10-fold in PI medium and added to Vero cells. After 72 h, the viral titers were determined as described above.

### Statistical analysis

All data were analyzed and plotted using GraphPad Prism (GraphPad Software). All error bars represent standard deviation (SD). The significance of the differences among groups in the cell-based study was determined using Student’s t test or one-way ANOVA with Tukey’s multiple comparison. A *p*-value of < 0.05 was interpreted as statistically significant. The results of the average daily gain (ADG) and fecal RNA shedding in the animal experiments were analyzed and the variables among groups were compared using a non-parametrical Kruskal–Wallis Test, with *p *< 0.05 considered significant. All data were analyzed using GraphPad Prism software (GraphPad Prism Inc.)

## Results

### Extraction and quantification of B. licheniformis-fermented products (BLFP) crude extract

**Fig. 1:**
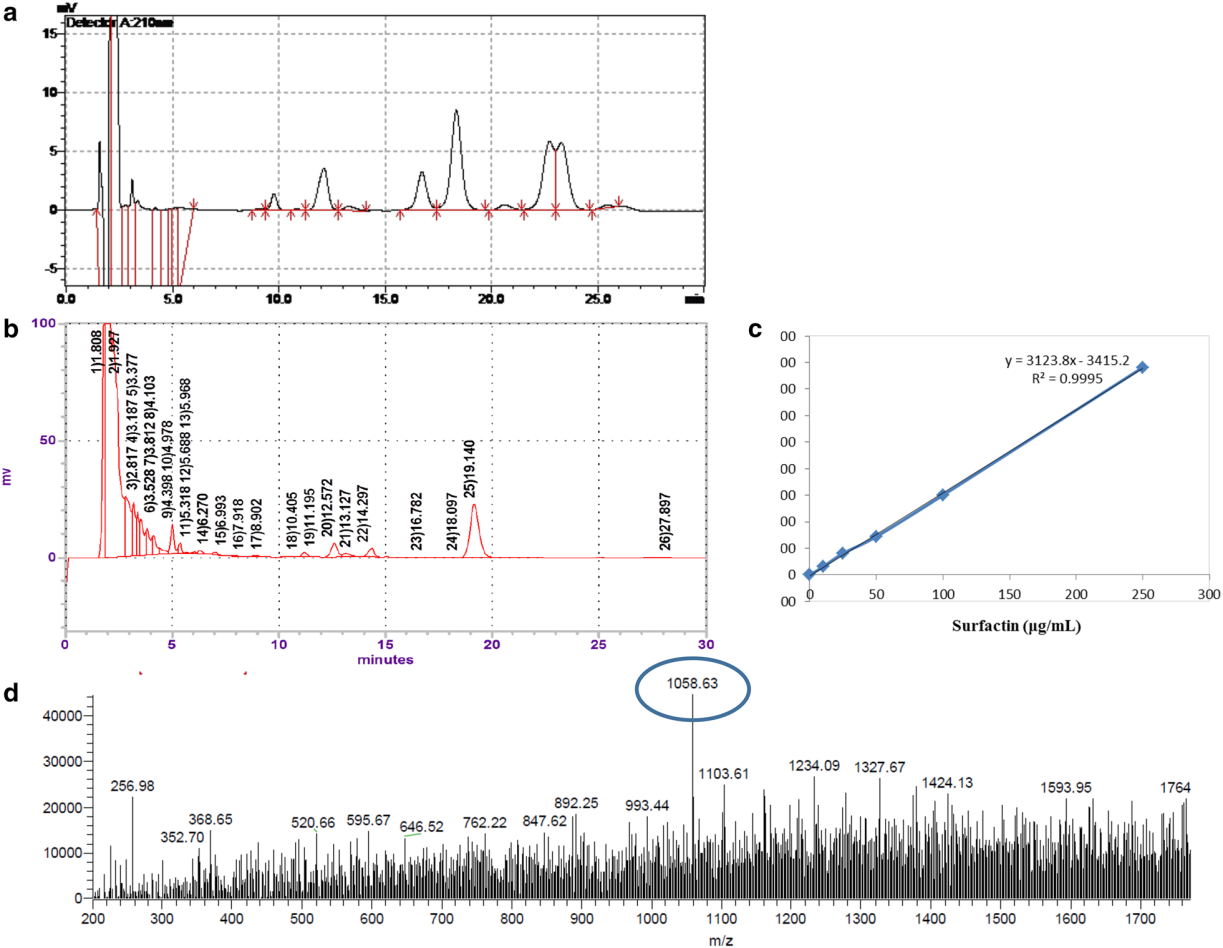
Identification of the 2nd metabolites in standard substance surfactin derived from *Bacillus subtilis* (**a**) and fermentative sample derived from *Bacillus licheniformis*-fermented products (BLFP) (**b**) by liquid chromatography–mass spectrometry (LC–MS). Using the standard curve of surfactin derived from *Bacillus subtilis* (**c**), the 2nd metabolite at [M+Na]^+^*m/z* 1058 in *Bacillus licheniformis*-fermented products crude extract (**d**) was identified and quantified

As shown in Fig. 1, HPLC analysis of the *B. licheniformis* extract showed a single peak (Fig. 1b) at the retention time of 16–17 min, which is identical to the standard substance surfactin derived from *Bacillus subtilis* (Fig. 1a). LC–MS analysis revealed that the 2nd metabolites derived from *B. licheniformis* fermentative cultivates appeared at [M+Na]^+^*m/z* 1058.35, suggesting a molecular weight of 1036 Dalton (Da) (Fig. 1).

### Clinical scoring of fecal consistency

**Fig. 2:**
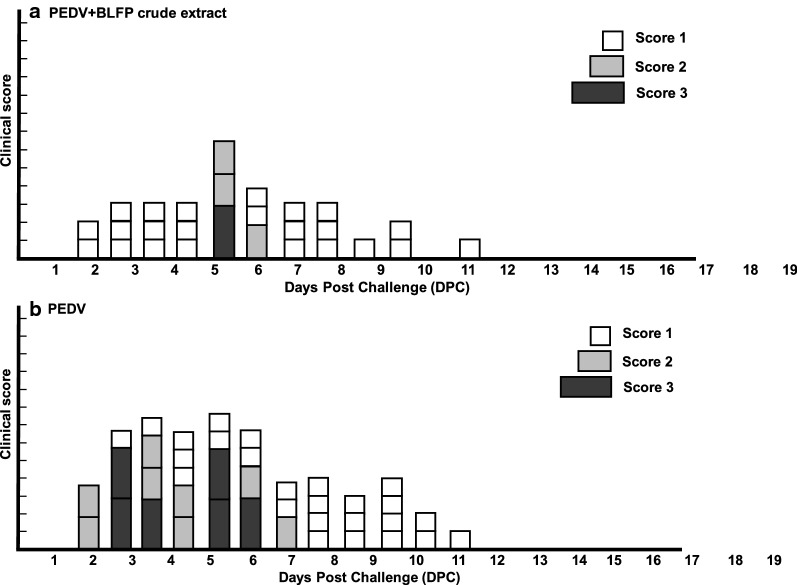
Clinical scoring of fecal consistency. Compared to PEDV-infected groups, the appearance and duration of typical diarrhea (score 2–3) were reduced in the PEDV + BLFP group

To assess the efficacy of the antiviral ability of BLFP against PEDV, clinical symptoms after viral challenge were recorded daily (Fig. 2). The fecal conditions of pigs in all groups were scored as normal (Score = 0) before PEDV inoculation. In general, pigs supplemented with BLFP exhibited milder symptom compared to piglets supplemented with controls. In the control group, all five pigs (5/5) exhibited normal clinical signs (Score = 0) during the study. In the PEDV group in which pigs were supplemented with control food, different severities of PEDV-associated clinical signs were first detected 2 days post infection (DPI). Two of these five pigs (2/5) exhibited semi-fluid feces (Score = 2) at 2 DPI, while 3/5 pigs showed typical semi-fluid (Score = 2) to watery diarrhea (Score = 3) at 2 to 8 DPI, and eventually recovered after 8 DPI. In the PEDV + BLFP group, most of the pigs exhibited pasty feces (Score = 1) at 2–5 DPI. Only one pig exhibited transient typical watery diarrhea (Score = 3) at 6 DPI. Compared to the PEDV-infected group with control food, the severity and duration of typical diarrhea (scores 2–3) were reduced in the PEDV + BLFP group.

### Fecal PEDV viral RNA

**Fig. 3:**
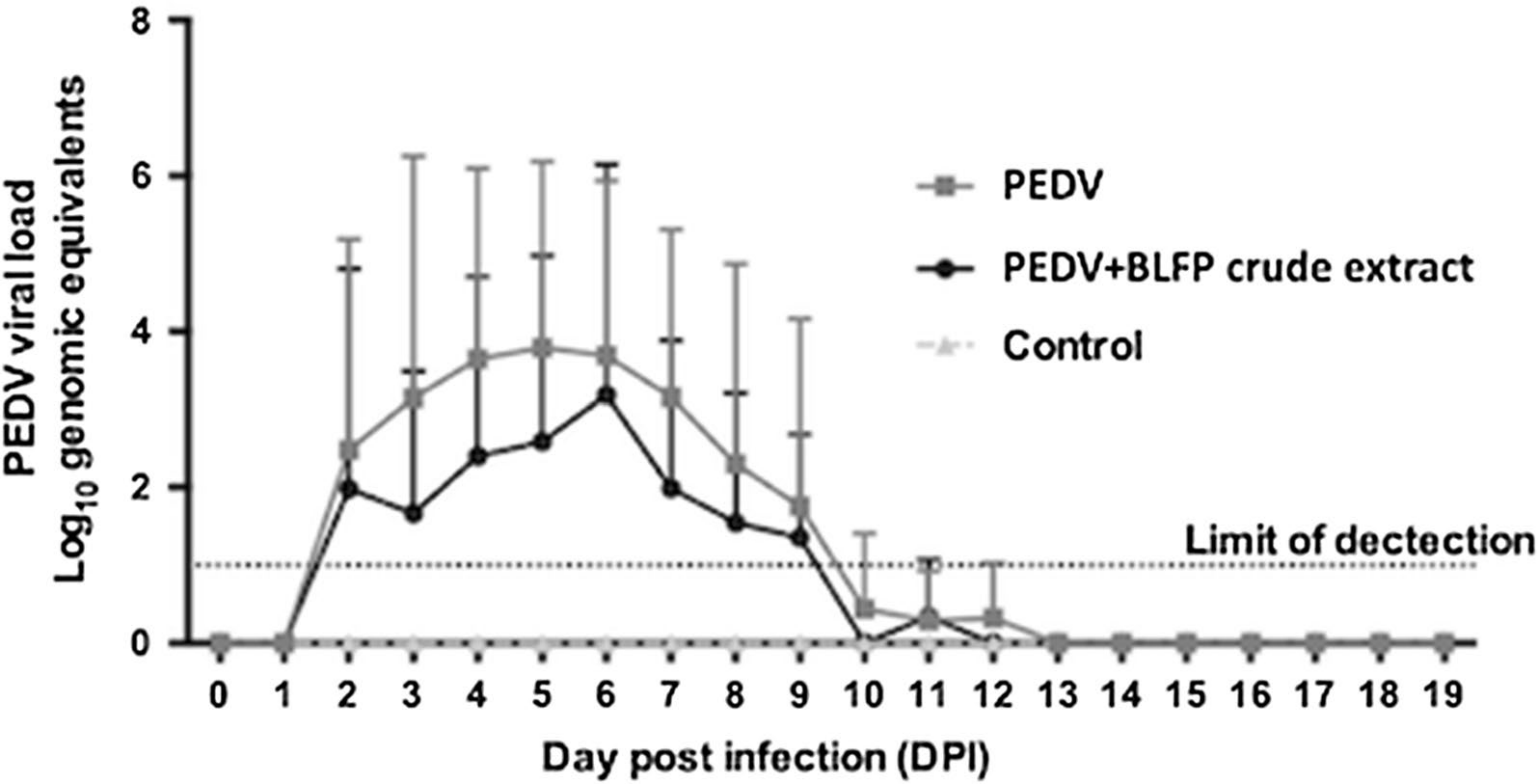
Fecal shedding of PEDVPT-P6 detected in piglets fed < 5 kg/ton BLFP in each group (n = 5). The pattern of viral shedding in the PEDV + BLFP group was similar to but lower than that of the PEDV group during the study, with no significant difference detected. Changes in the mean values of genomic equivalents (GE)/mL are presented as log10 values ± SD. The viral RNA loads of inoculated groups and the control group were compared using a non-parametrical Kruskal–Wallis test

To quantify PEDV-associated fecal viral shedding to evaluate the antiviral efficacy of BLFP against PEDVPT-P6, RT-qPCR was used to detect fecal viral shedding. In the PEDV group (the grey line in Fig. 3), fecal viral shedding was first detected (3.92 ± 3.88 log_10_GE) at 2 DPI, found to gradually increase to peak viral load (5.71 ± 3.38 log_10_GE) at 5 DPI, and was continuously detected until 12 DPI. In the PEDV + BLFP group (the black line in Fig. 3), the pattern of viral shedding was similar to but lower than that inthe PEDV group during the study, although the difference was not statistically significant.

### Growth performance of pigs supplemented with BLFP

**Fig. 4:**
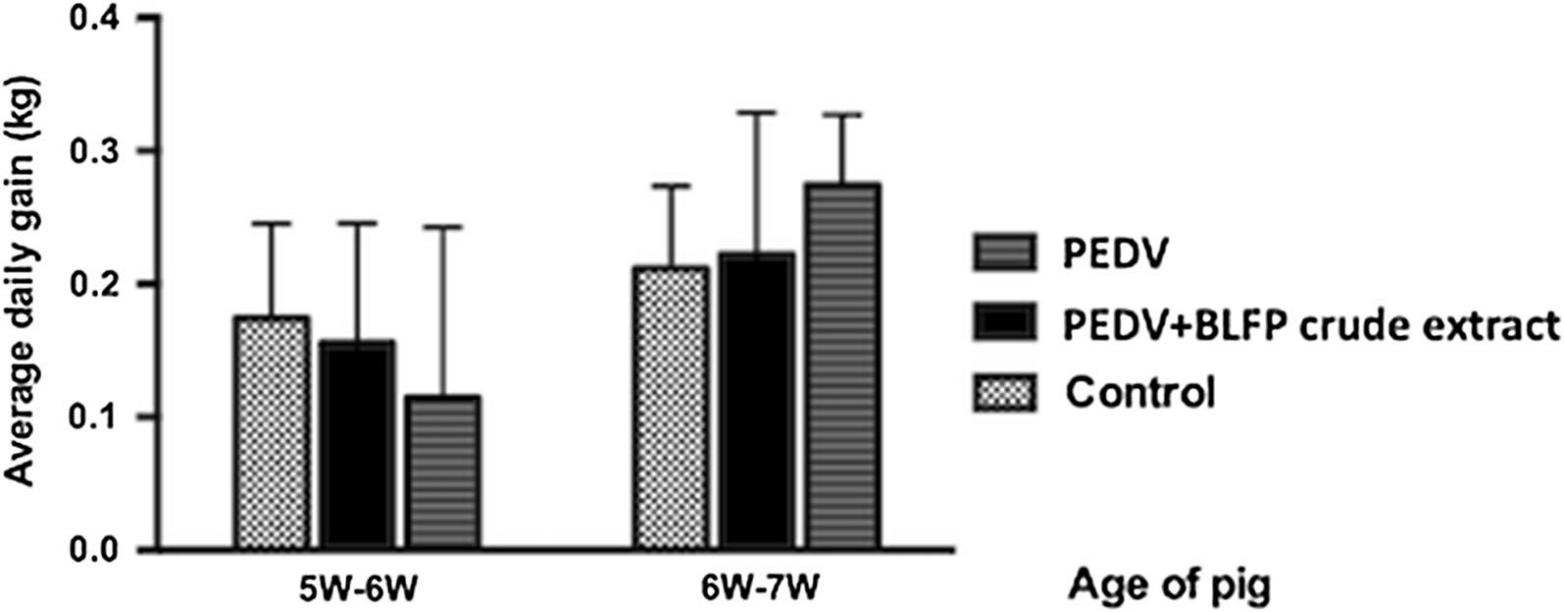
Average daily gain of pigs in each group. All pigs were weighed weekly in order to evaluate their growth performance with or without BLFP as feed additives. No statically significant difference in the average daily gain was noted among all groups each week

All pigs were weighed weekly in order to evaluate their growth performance with and without biosurfactants as a feed additive. No significant difference in the average daily gain (ADG) of pigs was noted among all groups in each week (Fig. 4).

### Gross and histopathological evaluation

To evaluate the safety of BLFP as a feed additive for pigs, necropsy was performed in all piglets 3 weeks post-infection for gross and histopathological examination. For the gross and histopathological examinations, no macroscopic or microscopic lesions were noted in any of the groups. These findings suggest that BLFP as a feed additives at 5 kg/ton are safe in conventional pigs up to 26 days of feeding.

### The median cytotoxic concentration (CC_50_) of BLFP crude extract on Vero cells

**Fig. 5:**
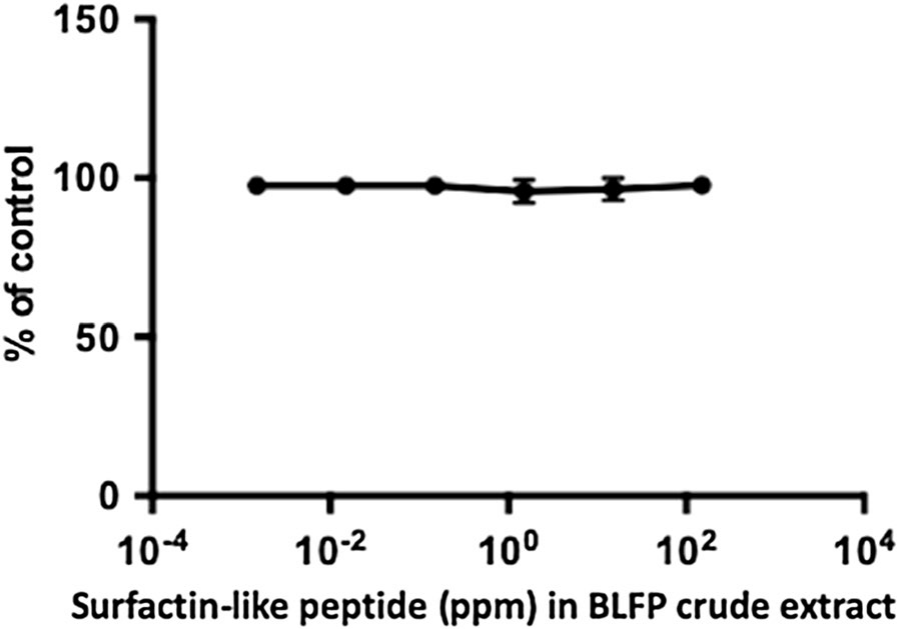
The 50% cytotoxicity concentration of crude extract derived from *Bacillus licheniformis* (BLFP) in Vero cells. Different concentrations of BLFP crude extract were added to Vero cells and incubated for 48 h. BLFP crude extract showed no cytotoxicity to Vero cells when evaluated using the AlamarBlue™ assay. Data are presented as the mean ± SD out of three test replicates

To determine the median cytotoxic concentration (CC_50_) of BLFP crude extract in Vero cells, the BLFP crude extract was serially diluted 10-fold from 150 to 0.015 ppm and added to cells. 48 h post-incubation (HPI), no cytotoxicity due to BLFP crude extract was observed in Vero cells (Fig. 5).

### Maximal inhibitory concentration (IC_50_) determination

**Fig. 6:**
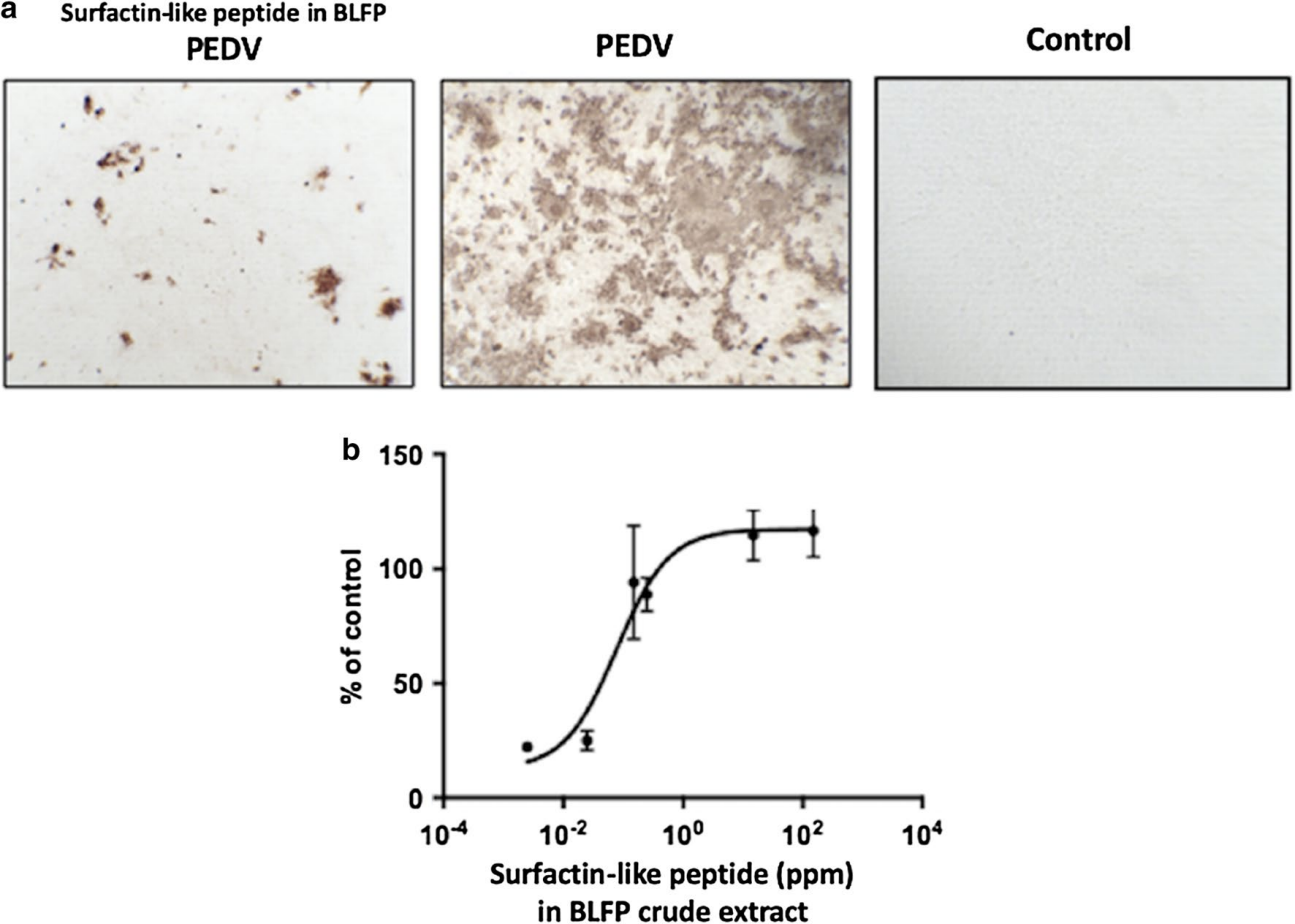
Antiviral activity of crude extract derived from *Bacillus licheniformis* (BLFP) against PEDV infection, as well as the IC_50_, was determined using the AlamarBlue™ assay. **a** PEDV-infected Vero cells treated with (left) and without (middle) 150 ppm BLFP crude extract. The right panel shows Vero cells without any treatment. Brown coloration indicates a positive PEDV S protein signal located in the cytoplasm of PEDV-infected cells. **b** Dose-dependent effect of BLFP crude extract against PEDV. The half-maximal inhibitory concentration (IC_50_) of BLFP crude extract against PEDV in Vero cells is 0.07 ± 0.45 ppm. Data are presented as the mean ± SD out of three test replicates

To determine the maximal inhibitory concentration, the post-drug group G6 was used to evaluate the maximal inhibitory effect of BLFP crude extract by cocultivating BLFP crude extract with PEDV-infected cells during the whole study. The results indicated that the antiviral activity of BLFP crude extract is dose-dependent, and the inhibition of PEDV-induced CPE was calculated with an IC_50_ value of 0.07 ± 0.45 ppm (Fig. 6).

### Time-of-addition assay of the BLFP crude extract and dose-dependent inhibition

**Fig. 7:**
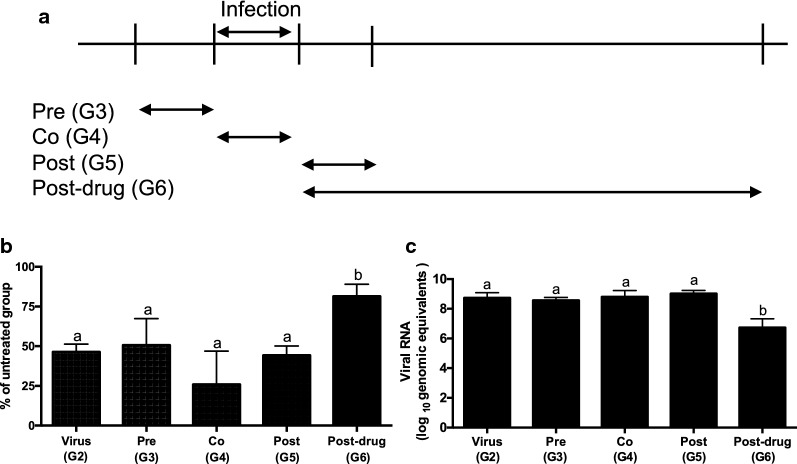
Time-of-addition assay of the BLFP crude extract. **a** Plot of the time-of-addition assays for BLFP crude extract treatment. 150 ppm of BLFP crude extract was added to PEDV-infected cells with various treatments. **b** Evaluation of antiviral activity of BLFP crude extract by AlamarBlue™ assay. **c** Detection of viral RNA of PEDV in the supernatants. Statistical analysis was performed by one-way ANOVA and followed by Tukey’s multiple comparisons test. **a**, **b** significant differences between groups are indicated with different letters (*p *< 0.05)

To elucidate the antiviral activity of BLFP crude extract against PEDV, 150 ppm of BLFP crude extract was added to the culture at different time points during the viral infection. As shown in Fig. 6a, typical PEDV-induced CPE was characterized by formation of syncytial cells, cell death, and detachment from the culture plate in PEDV-infected cells. The percent reductions of AlamarBlue staining in G3–G5—cells treated with BLFP crude extract 1 h before infection, at the same time as infection, and 1 h after viral infection, then replaced with fresh PI medium—were calculated as 50.7 ± 16.2%, 26.0 ± 20.1%, and 44.3 ± 5.8%, respectively, and showed no significant differences from those in the PEDV-infected group G2 (Fig. 7b). For the post-drug group G6, significant inhibition of PEDV-induced CPE, with an 81.5 ± 7.61% reduction of AlamarBlue staining, was observed compared to that in G2 and other groups (G3–G5). Similarly, the amount of viral RNA in supernatants from G6, ranging from 6.26 to 7.39 log_10_ GE, was significantly lower than that of G2–G5, ranging from 8.41 to 9.26 log_10_ GE (Fig. 7c). Additionally, the viral titer of supernatants in the G6 group, which was less than 10 TCID_50_/ml, was lower than that of G2–G5, ranging from 1 × 10^4^ to 1 × 10^5^ TCID_50_/ml. Moreover, when evaluating the direct virucidal activity of BLFP crude extract against PEDV, a reduction of the viral titer of PEDV from 10^5^ to 10^4^ TCID_50_/ml was observed.

### Replication kinetics of PEDV in cells with or without BLFP crude extract

**Fig. 8:**
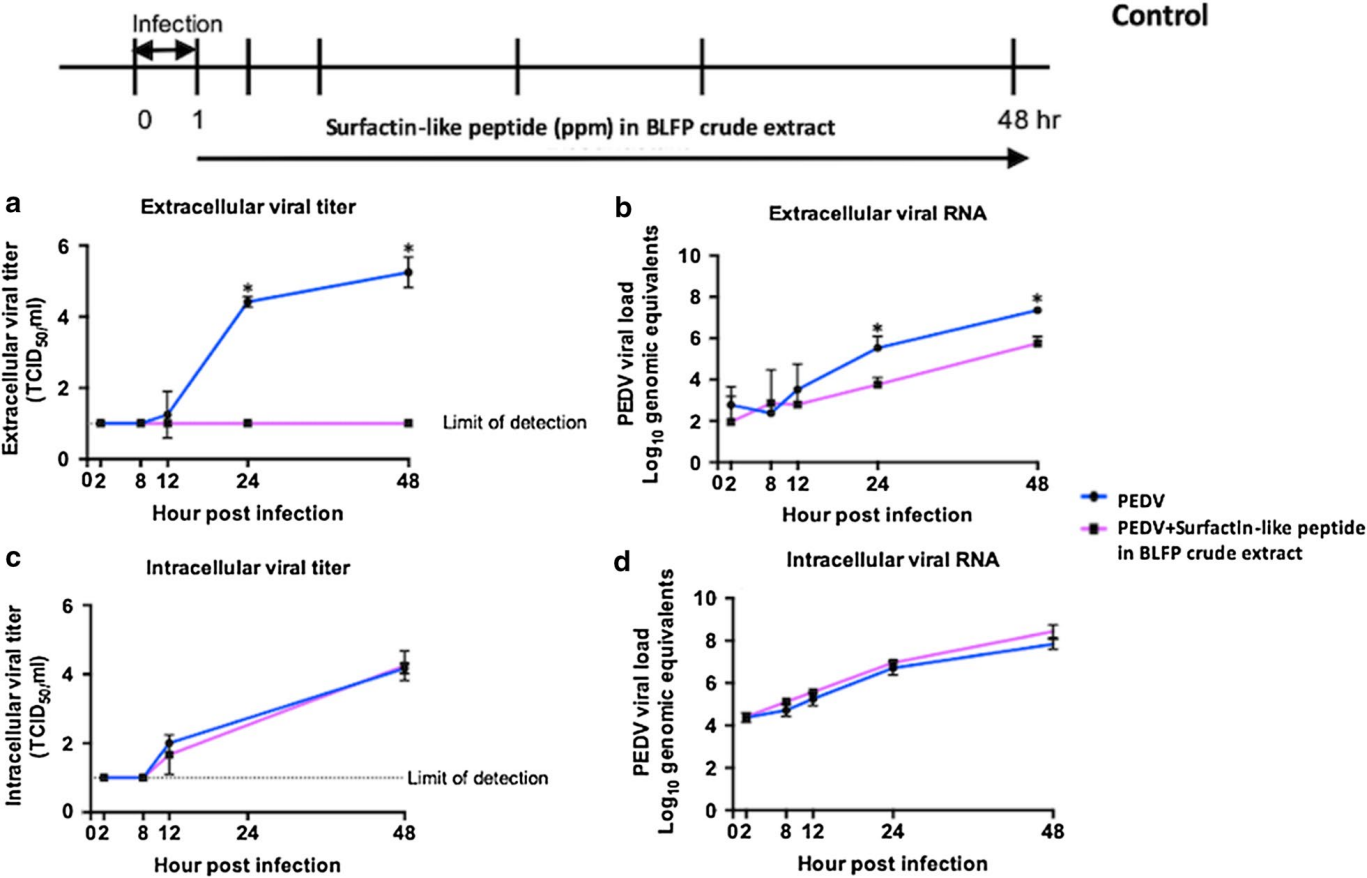
Replication kinetics of PEDV in Vero cells treated with or without BLFP crude extract. **a** Extracellular viral titers in the supernatants of PEDV-infected Vero cells treated with and without BLFP crude extract were determined by viral titration in Vero cells using the Reed-Müench method and expressed as the 50% TCID_50_/ml. **b** Extracellular viral RNA levels in the supernatants of PEDV-infected Vero cells treated with or without BLFP crude extract were determined by real-time reverse transcription (RT)-PCR. **c** Intracellular viral titers in PEDV-infected Vero cells treated with or without BLFP crude extract were determined by viral titration in Vero cells. **d** Intracellular viral RNA levels in the supernatants of PEDV-infected Vero cells treated with or without BLFP crude extract were determined by real-time RT-PCR. Statistical analysis was performed using Student’s t-test, and statistically significant differences were labeled with *(*p *< 0.05)

To further investigate the antiviral mechanism in the post-drug group (G6), the replication kinetics of PEDV in Vero cells in the presence or absence of BLFP crude extract were determined. Compare to PEDV-infected Vero cells cultured without BLFP crude extract, which showed viral titers from 1.78 × 10^5^ at 24 HPI to 3.16 × 10^6^ TCID_50_/ml at 48 HPI (Fig. 8a), cells treated with BLFP crude extract had an undetectable extracellular viral titer, which was significantly lower than that of cells without BLFP crude extract. Similarly, extracellular viral RNA levels in PEDV-infected cells cultured with biosurfactants were significantly lower than those without BLFP crude extract 24 and 48 HPI (Fig. 8b). However, no significant differences in intracellular viral titers (Fig. 8c) or intracellular viral RNA (Fig. 5d) were noted in PEDV-infected cells treated with or without BLFP crude extract.

## Discussion

Since late 2013, new variants of PEDV have been identified in several counties and have caused a devastating disease and economic loss. This viral threat has prompted a global effort to develop antivirals against new variant PEDVs in vitro (Song and Choi 2011; Kwon et al. 2013) and in vivo (Cho et al. 2012; Kim et al. 2015). Herein, we demonstrated that: (1) BLFP crude extract presents no cell toxicity to Vero cells, and exhibits significant antiviral ability against PEDVPT-P6 in Vero cells when biosurfactants are co-cultivated with PEDV; (2) Piglets supplemented with BLFP at concentrations of 5 kg/ton or less exhibited milder clinical symptoms and lower viral shedding compared to piglets supplemented with control food, and BLFP caused no significant toxicity. Therefore, BLFP is suggested to be a promising novel feed additive that can act as an additive against PEDV in the field.

The main antiviral mechanism of BLFP crude extract is thought to be related to its unique biochemical structure that increases membrane permeability to disrupt and lyse microbial membranes (Huang et al. 2006). By performing a direct-virucidal study of BLFP crude extract against PEDV, a ten-fold reduction of the viral titer was able to confirm the directed-antiviral ability of BLFP crude extract. According to our HPLC analysis, a peak of surfactin-like peptide was identified in the BLFP crude extract. Several different potential antiviral mechanisms of biosurfactants have been previously reported (Vollenbroich et al. 1997; Wang et al. 2017). Lipopeptide biosurfactants have been previously suggested to possess probiotic attributes for animal and human use (Schneider 1998; Sen 2010). A previous study demonstrated that the antiviral ability of surfactin from *B. subtilis* was achieved through competition with the TGEV entry receptor (Wang et al. 2017). The inhibition of viral enzymes such as proton-ATPase that are required for the entry of some viruses into cells by surfactin from *B. subtilis* (Vollenbroich et al. 1997) has also been reported. It is worth noting that not only did co-cultivation of the surfactin-like peptide in the BLFP crude extract with PEDV-infected Vero cells significantly reduce viral infection and replication in the study, but BLFP crude extract-treated PEDV-infected cells also exhibited an undetectable extracellular viral titer, suggesting that BLFP crude extract may play an important role in reducing the release of virions. These results suggest additional mechanisms of BLFP crude extract against PEDV in Vero cells. Although further study of the antiviral mechanisms of BLFP crude extract is needed in the future, observation of the diverse antiviral activities of BLFP crude extract will lead to further development of this new therapeutic candidate.

Previous studies demonstrated that lipopeptides produced by various *Bacillus* spp. were safe in mice (Youn-Hwan Hwang et al. 2008) and weaned piglets (Torres et al. 2017) when administered under a certain concentration. In order to use BLFP as a feed additive for its antiviral properties, assessment of its antiviral and safety profile in pigs is essential. Our results demonstrated that piglets supplemented with BLFP show milder symptoms and have reduced viral shedding. Importantly, no significant systemic pathological effects and no changes in average daily gain were noted. These results suggest that the biosurfactants at concentrations of 5 kg/ton or less will be very safe in pigs in future applications. In a previous study, it was found that administration of surfactin from *B. subtilis* at over 500 kg/ton leads to necrosis of hepatocytes in rats (Hwang et al. 2009). In the future, animal experiments using higher dosages of biosurfactants would be necessary to validate its maximal safety dosage.

In the present study, BLFP was investigated as an antiviral candidate against PEDV. In vitro, BLFP crude extract exhibited antiviral activities against PEDV when incubated long-term with PEDV-infected cells. In vivo, we first validated the safety and antiviral ability against of BLFP as a feed additive less than 5 kg/ton against PEDV after a long period of PEDV inoculation. Our results suggest that BLFP could be a novel candidate feed additive for reducing the titer of PEDV in the swine industry.

## Data Availability

The datasets used and/or analyzed during the current study are available from the corresponding author on reasonable request.
